# CDK1 promotes nascent DNA synthesis and induces resistance of cancer cells to DNA-damaging therapeutic agents

**DOI:** 10.18632/oncotarget.21730

**Published:** 2017-10-10

**Authors:** Hongwei Liao, Fang Ji, Xinwei Geng, Meichun Xing, Wen Li, Zhihua Chen, Huahao Shen, Songmin Ying

**Affiliations:** ^1^ Department of Respiratory and Critical Care Medicine of Second Affiliated Hospital, Zhejiang University School of Medicine, Hangzhou, China; ^2^ Department of Pharmacology, Zhejiang University School of Medicine, Hangzhou, China

**Keywords:** CDK1, DNA replication, chemotherapy resistance, DNA damage response, PARP

## Abstract

Cyclin dependent kinase 1 (CDK1) is essential for cell viability and plays a vital role in many biological events including cell cycle control, DNA damage repair, and checkpoint activation. Here, we identify an unanticipated role for CDK1 in promoting nascent DNA synthesis during S-phase. We report that a short duration of CDK1 inhibition, which does not perturb cell cycle progression, triggers a replication-associated DNA damage response (DDR). This DDR is associated with a disruption of replication fork progression and leads to genome instability. Moreover, we show that compromised CDK1 activity dramatically increases the efficacy of chemotherapeutic agents that kill cancer cells through perturbing DNA replication, including Olaparib, an FDA approved PARP inhibitor. Our study has revealed an important role for CDK1 in the DNA replication program, and suggests that the therapeutic targeting CDK1 may be a novel approach for combination chemotherapy.

## INTRODUCTION

Control of the cell division cycle is a fundamental process required by all proliferating cells. Cell cycle progression is driven by cyclin-dependent kinases (CDKs), whose activities are precisely regulated by their association with cyclins. The various cyclins present in human cells are expressed and degraded in a manner that is strictly coordinated with cell cycle progression [1]. In lower organisms, such as yeast, CDK1 (also referred to as Cdc2) is the only CDK required to promote cell cycle progression through its sequential binding to different cyclins. However, in mammalian cells, there are multiple CDKs and cyclins that can form a diverse array of CDK-cyclin complexes acting at different stages of the cell cycle [2].

The accepted paradigm for cell cycle regulation in human cells by CDKs has been as follows: (i) CDK4 and CDK6 promote G1 progression through binding to cyclin D. (ii) The G1 to S transition and the initiation of replication are driven by an association of CDK2 with cyclin E and cyclin A, respectively. (iii) CDK1 associates with cyclin B to activate entry into mitosis and regulates several aspects of mitotic progression [3-6]. However, this paradigm has been challenged somewhat during recent years by the emergence of multiple lines of evidence indicating significant redundancy among CDKs. Normally individual loss of one CDK is not lethal because its function can be compensated by the remaining CDKs [7]. But there is one exception, CDK1, loss of which is embryonic lethal [8]. CDK1 appears to be predominant comparing to other CDKs, which can be reflected by the interrelationship between CDK1 and CDK2. CDK1 has the capacity to execute all the essential CDK2 functions when CDK2 is absent, but not vice versa [8, 9]. The capability of CDK1 to compensate for CDK2 loss is reminiscent of the fact that CDK1 is the only CDK in lower organisms, which suggests that the versatile nature of CDK1 could be masked due to the presence of other CDKs. Because CDK1 overexpression is often seen in human diseases such as cancer [10-12], a better understanding of its functions will be of great therapeutic relevance.

Over the past decade, much progress has been made in characterizing the multifaceted role of CDK1. Studies showed that CDK1 could drive G1/S transition by associating with cyclin E, which was thought to be an exclusive CDK2-binding partner [9]. It was also reported that in the absence of CDK2, CDK1 activity is essential for centrosome duplication [13]. Moreover, a recent study indicated that CDK1 was required for the faithful completion of replication in late S phase, by competitively inhibiting the formation of CDK2-cyclin A complexes and therefore suppressing re-replication of already duplicated DNA [14]. Besides these functions, CDK1 was reported to participate in homologous recombination (HR)-dependent repair of DNA double-strand breaks (DSBs) and in checkpoint activation through functional phosphorylation of BRCA1 [15-17], and to couple DNA damage repair pathways to cell cycle progression [18-20]. Collectively, CDK1 presents itself as a master regulator of multiple core biological events including cell cycle progression, DNA replication, and DNA damage repair.

In this article, we have uncovered another unanticipated role for CDK1 in promoting nascent DNA synthesis and DNA replication fork progression, even in cells expressing CDK2. We show that a short duration of CDK1 inhibition induces a significant DNA damage response, which is associated with DNA replication. Further, we show that CDK1 inhibition sensitizes cancer cells to different chemotherapeutic agents.

## RESULTS

### CDK1 inhibition induces a DNA damage response

**Figure 1:**
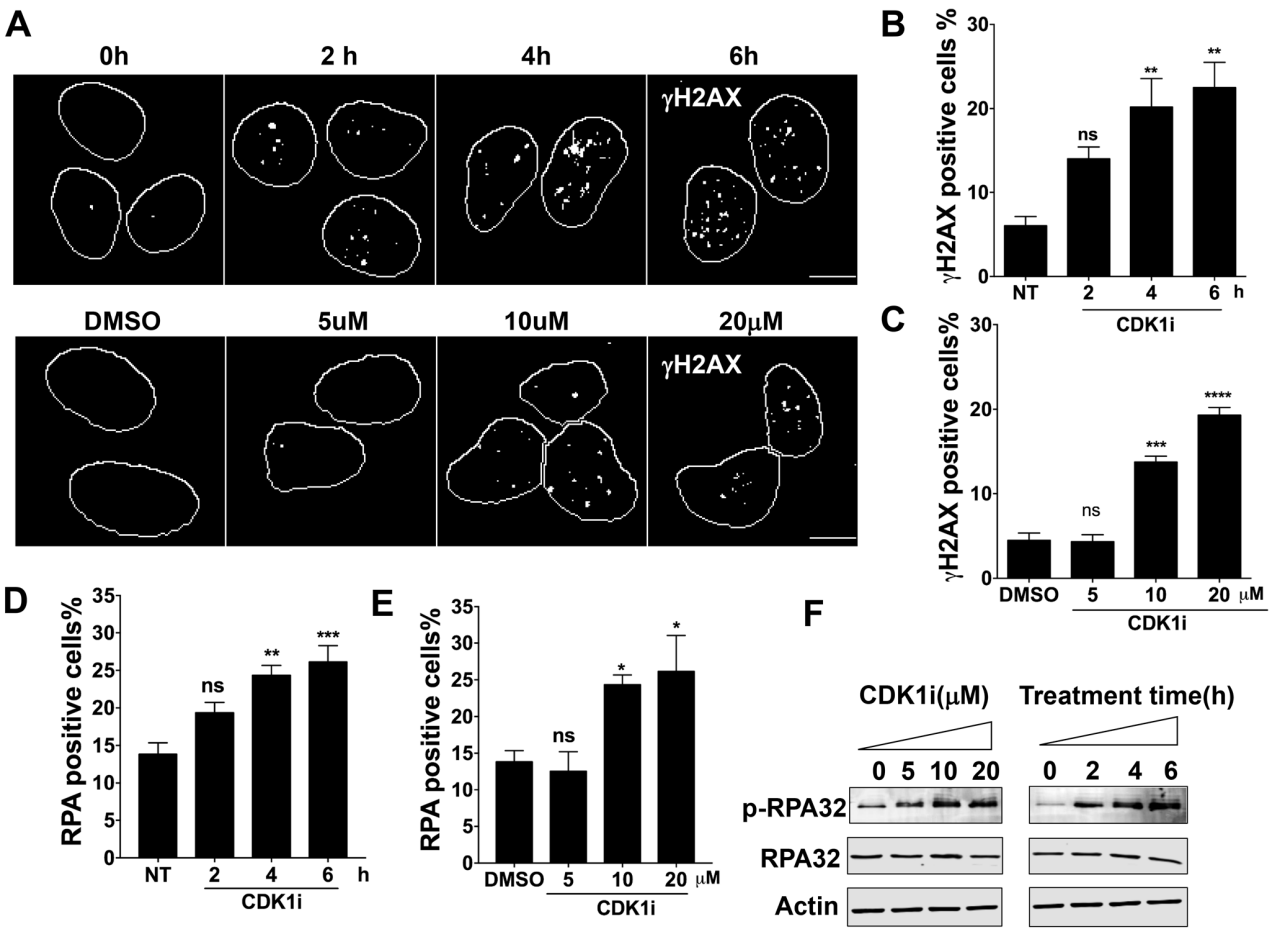
Short-term exposure to CDK1 inhibitors leads to a DNA damage response in a time- and dose-dependent manner **A.** Representative images of γH2AX foci formation in U2OS cells treated with 10 µM CDK1 inhibitor (CDK1i) for the indicated times (upper panel) or with increasing concentrations of CDK1i for 4 hours (lower panel); DMSO, dimethyl sulfoxide; scale bar, 20 µm; **B.**-**C.** Quantification of the data from the upper and lower panels of A. Cells with over 5 γH2AX foci were counted as positive cells; **D.**-**E.** Quantification of RPA foci in U2OS cells treated with 10 µM CDK1i for the indicated times **D.** and in U2OS cells exposed to increasing concentrations of RO3306 for 4 hours **E.**. Cells having more than three RPA foci were considered to be RPA positive; **F.** Immunoblot analysis for indicated proteins in the whole cell extracts from U2OS cells treated as graphed. Actin was used as a loading control. Data are from three independent experiments and are presented as the means±SEM. To determine significance, a one-way ANOVA followed by Dunnett’s multiple comparison post-test was performed for B-E. A Student’s *t*-test was performed: *, *p* < 0.05; **, *p* < 0.01; ***, *p* < 0.001; ****, *p* < 0.0001; ns, not significant.

CDK1 has been shown to participate in DNA damage repair, especially in HR-mediated repair of DSBs *via* a role in facilitating end resection [17, 18, 21]. We set out to investigate whether short-term exposure of cells to a CDK1 inhibitor might reveal additional roles in the DNA damage response (DDR). To this end, we exposed U2OS cells to a specific CDK1 inhibitor RO3306 [22]. We treated the cells for periods of time up to 6 hours. We observed the treatment triggered a significant DDR in a time- and dose-dependent manner, as indicated by the formation of γH2AX and RPA foci (Figure 1A-1E). We also detected RPA hyperphosphorylation, which is a marker of DDR activation (Figure 1F). To confirm that the effects could be reproduced in a cell line derived from a different organ, we analyzed A549 lung carcinoma cells and observed similar results (Supplementary Figure 1A-1C).

Next, we sought to identify the underlying cause of this cellular phenotype. Given the canonical role of CDK1 in regulating cell cycle progression, we first investigated whether the DDR caused by CDK1 inhibition was a result of an arrest of cell cycle progression. However, we observed that the CDK1 inhibitor had no significant impact on the cell cycle profile over a 6-hour period of exposure (Supplementary Figure 1D-1E). Because CDK1 also participates in HR-dependent DSB repair, compromised CDK1 activity might lead to an accumulation of unrepaired DSBs, which consequently activates DDR as we observed [16]. However, this explanation was also precluded, as we failed to observe any increase in the number of 53BP1 foci (a specific marker of DSBs) following CDK1 inhibition for up to 6 hours (Supplementary Figure 1F). After ruling out cell cycle regulation and DNA damage repair, we hypothesized that transient CDK1 inhibition-induced DNA damage is directly linked to DNA replication.

### CDK1 inhibition-induced DDR is DNA replication dependent

**Figure 2:**
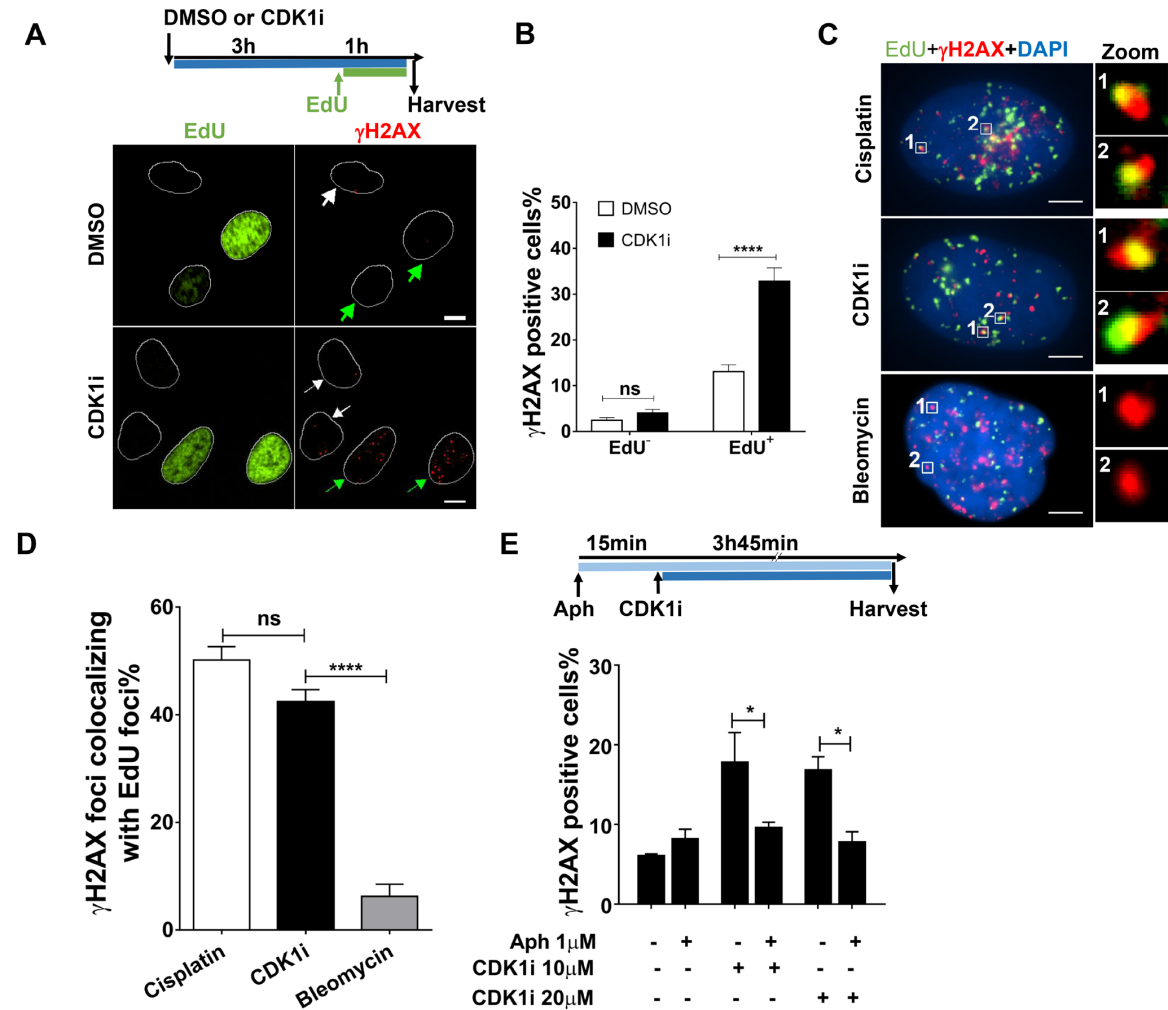
CDK1 inhibition induces a DNA damage response mainly in replicating cells **A.** Representative immunofluorescence images of γH2AX foci in DMSO- or CDK1i-treated U2OS cells. EdU staining was used to label replicating cells. White and green arrows denote non-replicating cells and replicating cells, respectively, and white circles are outlines of cell nuclei. An experimental scheme is shown at the top. Scale bar, 10 µm; **B.** Quantification of γH2AX foci in EdU-negative and -positive U2OS cells treated as indicated in **A. C.** Representative images of single cell-based analysis of colocalization of γH2AX foci and replication sites marked by EdU after the indicated treatment according to the scheme shown in A, insets on the right side are enlarged images of colocalization of γH2AX foci to EdU foci. Scale bar, 5 µm. **D.** Quantification of the percentage of γH2AX foci colocalizing with EdU foci in cells from **C.**, cisplatin and bleomycin were used as the positive and negative controls, respectively, and at least 30 individual cells were analyzed for each group. **E.** Quantification of γH2AX foci in U2OS cells treated with or without CDK1i in the absence or presence of aphidicolin. The experimental scheme is shown at the top. Data were obtained from three independent experiments and are presented as the means±SEM. To calculate significance, a two-way ANOVA followed by a Sidak multiple comparison post-test was used for **B.** and **E.**, and a one-way ANOVA followed by Dunnett’s multiple comparison post-test was performed for **D.** *, *p* < 0.05; ****, *p* < 0.0001; ns, not significant.

Because our data suggested that a short duration of CDK1 inhibition could give rise to replication-associated DNA damage, we sought to assess the contribution of DNA replication to CDK1 inhibition-induced DDR. First, we quantified the number of γH2AX foci in both replicating and non-replicating cells treated with or without a CDK1 inhibitor. EdU staining was used to mark replicating S-phase cells. We found that CDK1 inhibition predominantly caused a DDR in EdU-positive cells (Figure 2A-2B). Moreover, analysis of single cells revealed that a large proportion of the γH2AX foci co-localized with sites of ongoing DNA synthesis, as defined by EdU incorporation. Indeed, the co-localization percentage we observed was comparable with that seen in cells treated with cisplatin, which is a typical replication-dependent DNA-damaging agent that induces replication-blocking interstrand DNA crosslinks (Figure 2C-2D). In contrast, bleomycin, which chemically cleaves DNA strands, induced γH2AX foci that did not co-localize with sites of DNA synthesis (Figure 2C-2D). Importantly, when DNA replication was blocked by exposure to the DNA polymerase inhibitor, aphidicolin, the DDR normally activated by inhibition of CDK1 was abrogated (Figure 2E). Taken together, these results suggest that CDK1 plays a role in preventing replication-born DNA damage, thus ensuring efficient replication progression through S-phase.

### CDK1 activity is required for efficient DNA synthesis

**Figure 3:**
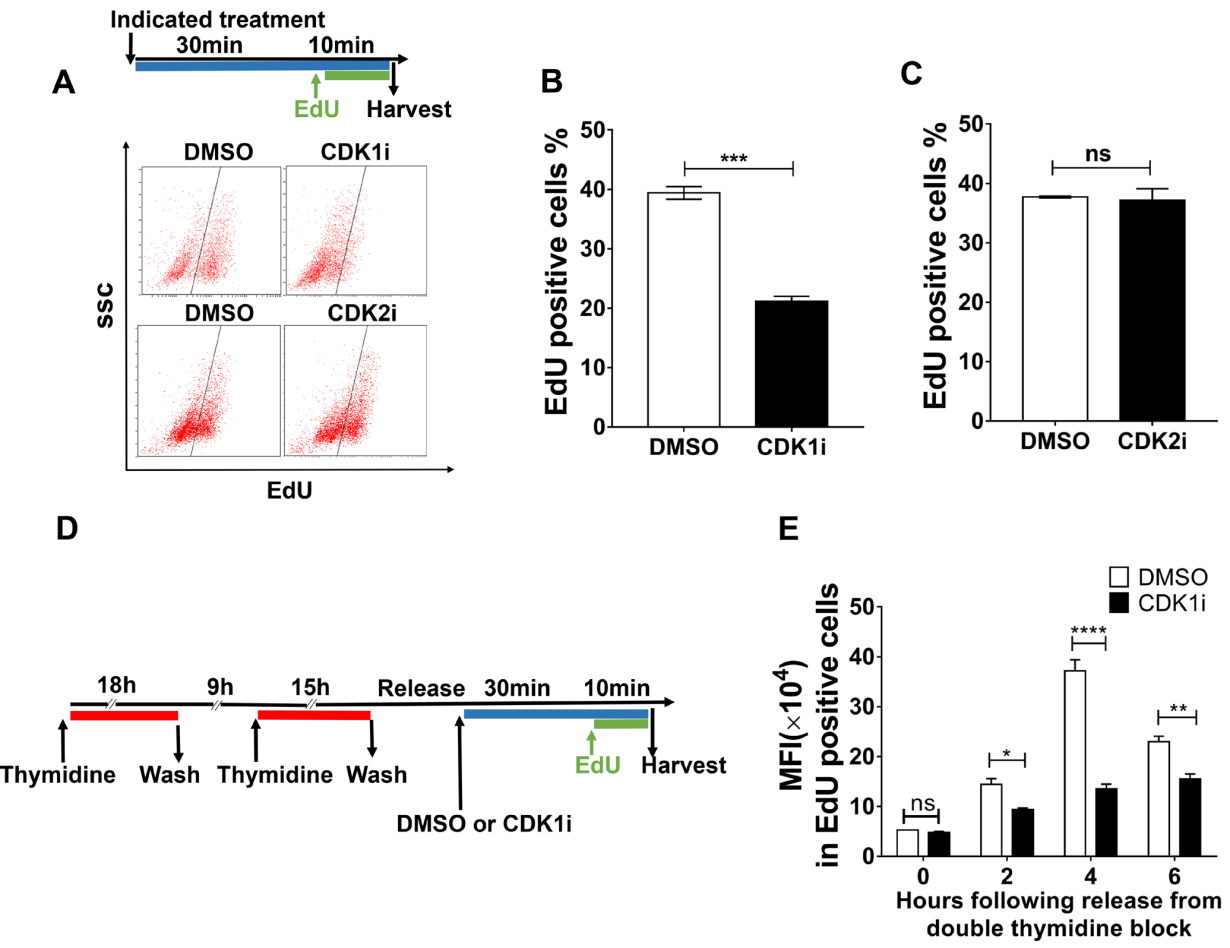
CDK1 activity is required to promote nascent DNA synthesis **A.** Representative FACS data from U2OS treated with the indicated inhibitors for 40 minutes and then pulse-labeled with EdU in the last 10 minutes of the incubation period. EdU-positive cells were gated as shown by the black line. The experimental scheme is shown at the top. **B.**-**C.** Quantification of EdU-positive cells gated from **A. D.** Workflow to determine the effects of CDK1i on DNA replication in different stages of S phase. **E.** Quantification of mean fluorescence intensity (MFI) in EdU-positive cells after the treatments applied according to the scheme shown in **D.** Data in **B.** and **C.** are pooled from three independent experiments and are presented as the means±SEM. Two independent experiments were conducted for **B.** and data are presented as the mean±SEM. A Student’s *t*-test was performed for **B.** and **B.** to determine significance. Multiple *t*-tests were performed for **E.** *, *p* < 0.05; **, *p* < 0.01, ****, *p* < 0.0001; ns, not significant.

To characterize the role of CDK1 in preventing replication-born DNA damage, we investigated whether it might act directly during the process of DNA replication. We performed fluorescence-activated cell sorting (FACS) to quantify nascent DNA synthesis revealed by EdU pulse labeling. We observed that CDK1 inhibition dramatically diminished EdU incorporation, and this finding was confirmed by microscopic detection of EdU foci (Figure 3A-3B, Supplementary Figure 2A-2B). Similar results were also observed in A549 cells, precluding the possibility of cell type specificity (Supplementary Figure 2C). Because CDK1 is known to facilitate the function of BRCA2, which was reported to maintain replication fork in a DSB repair-independent manner [23-25], we inferred that CDK1 might promote DNA replication through BRCA2. To test this hypothesis, we used a Chinese hamster ovary cell line VC8 which lacks BRCA2, and a VC8 cell derivative supplemented with the *BRCA2* cDNA (VC8+B2). We compared the effects of a short duration of CDK1 inhibition on EdU incorporation in those two cell lines. However, our results indicated that VC8 and VC8+B2 cells displayed a similar replication profile, and CDK1 inactivation caused a comparable reduction in the number of EdU-positive cells in those two cell lines (Supplementary Figure 2D-2E). These data suggested that CDK1 is unlikely to act in DNA replication *via* BRCA2.

We were intrigued by the observation that a reduction in EdU-positive cells could be observed following only a very short (10 mins) exposure to CDK1 inhibitors (Supplementary Figure 2F). This suggested that CDK1 might play a role directly in ongoing DNA synthesis. Because a reduction in nascent DNA synthesis could result from either insufficient replication origin firing or decreased replication fork progression, we investigated whether CDK1 inhibition could affect either origin usage or DNA replication fork speed. Given the overlapping functions of CDK1 and CDK2 in regulating origin firing, we first tested whether CDK2 inhibition could influence EdU incorporation as CDK1 inhibition did. However, we observed that CDK2 inhibitors could not exert an effect on EdU incorporation (Figure 3A, 3C), suggesting that the reduction in the number EdU-positive cells observed after CDK1 inhibitor treatment was not a result of a decrease in origin firing. To confirm this, we blocked cells at the G1/S boundary using a double thymidine block, released them synchronously into S phase, and then performed EdU pulse labeling at different time points (Figure 3D). We used EdU fluorescence intensity to quantify the effects of CDK1 inhibition on nascent DNA synthesis during the different stages of S-phase. We observed that CDK1 inhibitor treatment caused a significant decrease in EdU fluorescence intensity at all time points analyzed, but in particular at 4 hours after release from the double thymidine block (Figure 3E). These data suggest that CDK1 is required for efficient nascent DNA synthesis throughout S-phase, and are not consistent with the proposal that CDK1 is held in an inactive state prior to mitosis through Wee1- and Myt1-mediated inhibitory phosphorylation before mitosis.

### CDK1 activity promotes DNA replication fork progression and prevents fork stalling

**Figure 4:**
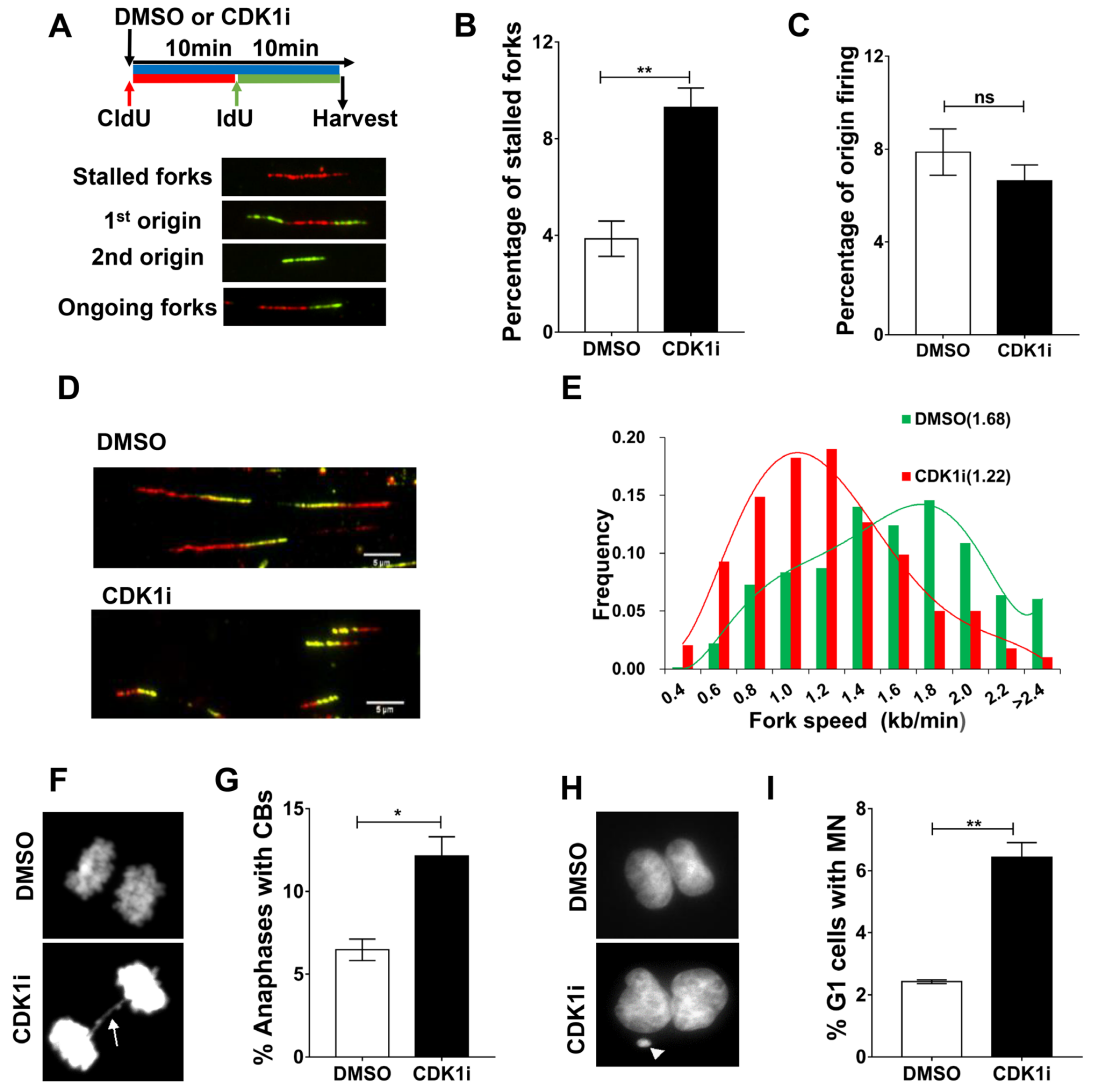
CDK1 inhibition leads to increased replication fork stalling and elevated genomic instability **A.** Replication fork analysis of U2OS cells pulsed with the thymidine analogs chlorodeoxyuridine (CldU) and iododeoxyuridine (IdU) in the presence of DMSO or CDK1i. An experimental scheme for DNA fiber assay (**A.**, top), typical replication structures (**A.**, bottom) are shown. **B.**-**C.** Quantification of the percentage of stalled forks **B.** and new origin firing **C.** in cells from A. **D.** Representative images of ongoing replication forks in U2OS cells treated with or without CDK1i (scale bar, 5 µm). **E.** Quantification of fork speed (numbers in the parentheses denote average fork speed) in cells from D (*n* = 4 independent experiments). In B and D, results are presented as the means±SEM. **F.**-**G.** Representative images **F.** and quantification **G.** of anaphases containing chromosome bridges (CBs) in U2OS cells treated with DMSO or 7 μM RO3306 for 16 hours, the white arrow denotes a typical chromatin bridge. **H.**-**I.** Representative images **H.** and quantification **I.** of G1 daughter pairs containing micronuclei (MN) from cells treated as described in **F.** The white arrow denotes a representative micronucleus. Data are presented as the means±SEM from three independent experiments, and at least 1000 anaphases or G1 daughter pairs were counted. Student’s *t*-tests were performed to calculate p values. *, *p* < 0.05; **, *p* < 0.01; ns, not significant.

To better understand the role of CDK1 in DNA replication at a molecular level, we performed DNA fiber assays to directly visualize alterations in replication fork dynamics. Consistent with the FACS data, CDK1 inhibition led to an increase in replication fork stalling without having a significant impact on origin firing (Figure 4A-4C). Moreover, the speed of progression of individual replication forks was also slowed in the presence of a CDK1 inhibitor (Figure 4D-4E). This negative effect on fork speed was also in the VC8 and VC8+B2 cell lines (Supplementary Figure 3A-3B), consistent with the proposal that the effect of CDK1 is not mediated through BRCA2. Taken together, these data indicate that CDK1 is required for replication fork maintenance, and that loss of its activity can generate replication stress.

Because stalled forks under prolonged replication stress can be converted into DSBs by DNA structure-specific endonucleases [26], and given the action of CDK1 in checkpoint activation and DSB repair, we hypothesized that prolonged CDK1 inhibition might allow transmission of DNA damage from S-phase into mitosis. If so, this would be expected to give rise to chromosome segregation errors during anaphase, such as the formation of chromatin bridges and lagging chromatin, leading to micronucleus formation in the subsequent G1. Consistent with this hypothesis, we observed that CDK1 inactivation in S-phase significantly increased the frequency of chromatin bridges and micronuclei (Figure 4F-4I).

### CDK1 inhibition increases sensitivity of cancer cells to chemotherapeutic agents

**Figure 5:**
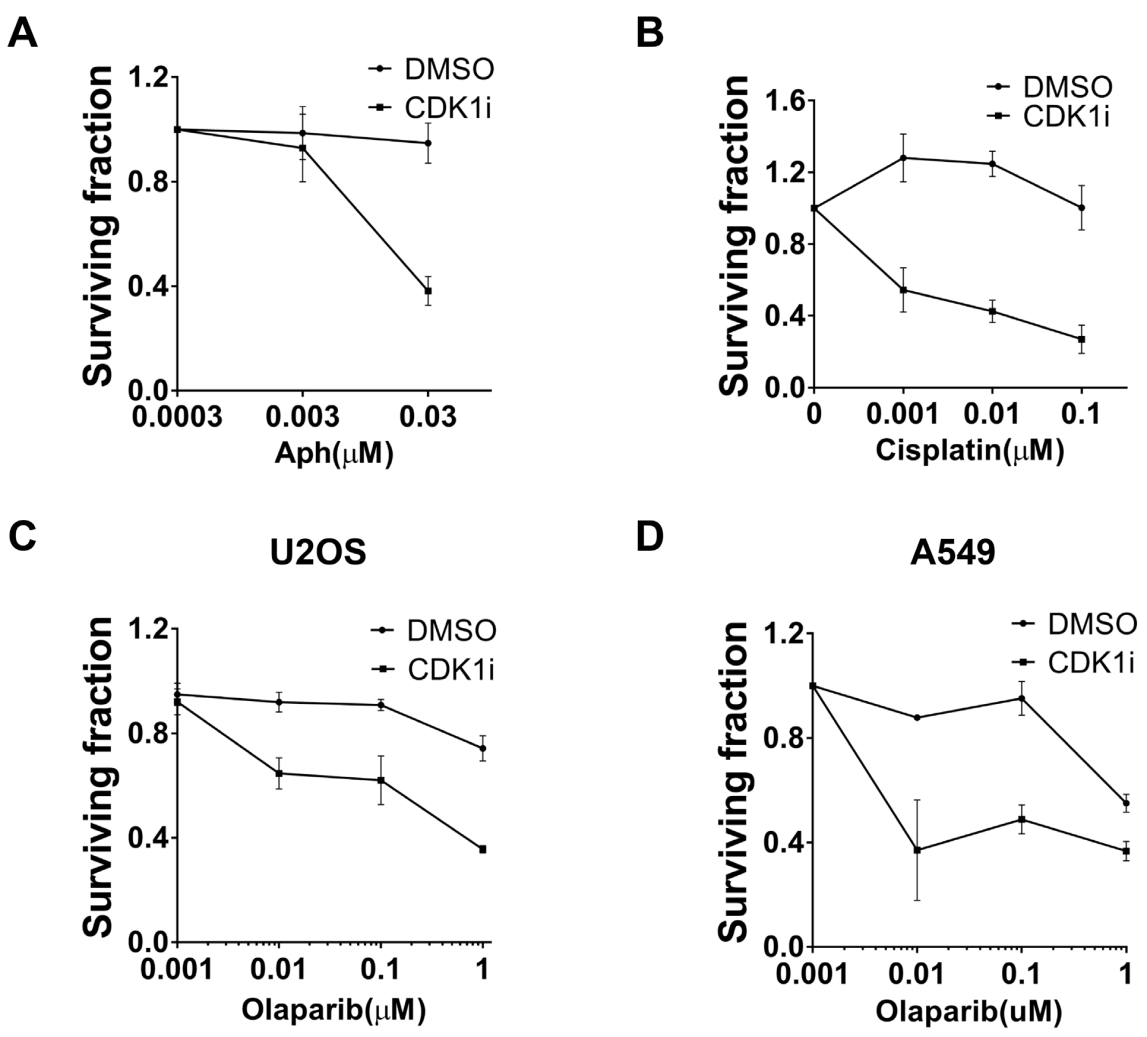
CDK1 inhibition resulted in increased sensitivity to multiple genotoxic agents **A.** Synergistic effects between aphidicolin (Aph) and CDK1 inhibitors. Clonogenic assays using U2OS cells treated with increasing concentrations of Aph in the absence or presence of 1 µM CDK1i were conducted. Colony numbers in each group (DMSO or CDKi) were scored as surviving fraction relative to cells without Aph treatment. **B.**-**C.** CDK1 inhibition increased the sensitivity of U2OS cells to clinically-used chemotherapeutic drugs including cisplatin **B.** and the PARP inhibitor, Olaparib **C.**. Cells were analyzed as indicated in **A**. **D.** A549 cells were treated with increasing concentrations of Olaparib in the absence or presence of 1 µM CDK1i. Data are presented as the mean±SEM of three independent experiments.

Based on our finding that CDK1 is essential for promoting DNA replication fork progression, we explored the possible therapeutic relevance of CDK1 inhibitors in combination with other chemotherapeutic agents in cancer treatment. To this end, we performed clonogenic survival assays. We observed that CDK1 inhibition sensitized cancer cells to different genotoxic agents, including aphidicolin and cisplatin (Figure 5A-5B). Notably, the newly FDA-approved PARP inhibitor, Olaparib, which is generally assumed to only show good efficacy in patients carrying a BRCA mutation [27], displayed enhanced cytotoxicity in BRCA-proficient U2OS and A549 cells in the presence of a CDK1 inhibitor (Figure 5C/5D). Thus, CDK1 inhibitors can potentially be used not only to potentiate the efficacy of chemotherapeutic agents, but also to extend the therapeutic spectrum of chemotherapeutic drugs.

## DISCUSSION

Our study has identified a previously unanticipated role for CDK1 in promoting efficient DNA replication. Inhibition of CDK1 elicited a DNA damage response and gave rise to genomic instability and impaired cell viability. Consistent with this pivotal function of CDK1, cancer cells displayed hypersensitivity to a range of DNA damaging agents when CDK1 activity was inhibited.

The mechanism underlying the regulation of replication fork progression by CDK1 requires further investigation, although it is likely to be a direct result of the ability of CDK1 to phosphorylate specific target proteins. Moreover, whether the CDK1 activity is necessary for all replication forks or merely required for a subset remains unclear. DNA replication is a complex, multifaceted process that depends crucially upon the proper functioning of replisome components, an adequate supply of dNTPs, a coordination with gene transcription, and efficient repair mechanisms that clear endogenous barriers to replication forks [28]. Given this complexity, it will be challenging to identify the specific downstream effectors of CDK1 that are directly involved in DNA replication. One plausible function of CDK1 activity is regulation of the nucleotide supply for replication because it is known that the deoxynucleotide triphosphohydrolase SAMHD1, a major regulator of the dNTP pool in mammalian cells, is a substrate of CDK1-cyclin A2 dependent phosphorylation [29, 30]. Inhibition of CDK1 did not completely abrogate DNA replication as HU does, suggesting that even if nucleotide pools are compromised by CDK1 inhibition, the effect must be milder than that seen following inhibition of ribonucleotide reductase by HU.

Because DNA replication is a highly complex and coordinated process involving many factors, it is difficult to individually examine how specific factors act without perturbing the whole process. Nevertheless, there is scope to identify the key “conductors” that are responsible for orchestrating the replication program. Our work provides the first clue that CDK1 is likely to be one of the factors that serve as a key regulator of DNA replication without being part of the machinery required for DNA synthesis per se. Further work will be required. Perhaps by using a combination of CDK1 inhibitors and phosphoproteomics, we can characterize how the DNA replication machinery is modified through regulatory phosphorylation by CDK1 to guarantee accurate and timely genome duplication.

The functional versatility of CDK1 is remarkable. Besides its role in cell cycle control, CDK1 also functions in DNA damage repair and checkpoint activation, and our work now reveals a role in DNA replication fork progression. Thus, CDK1 inhibition stimulates replication-associated DNA damage, compromises the repair mechanism, and allows transmission of unrepaired DNA damage into mitosis because of a failure to activate the appropriate checkpoint. A characteristic feature of cancer cells is uncontrolled proliferation that relies on efficient replication progression, despite the fact the oncogene activation frequently leads to constitutive replication stress in pre-neoplastic and neoplastic cells. As a result, cancer cells are particularly sensitive to DNA replication perturbation [31, 32]. A range of currently used chemotherapeutic drugs, such as cisplatin, exploit this vulnerability in cancer cells. However, because of the presence of efficient repair mechanisms in some cancer cells, the efficacy of these chemotherapeutic drugs is often limited. Therefore, the use of a combination of different chemotherapies is often a better therapeutic strategy and is widely used in clinical practice. Because of the multifaceted role of CDK1 in diverse cellular events that are vital for the survival of proliferating cells, we consider that CDK1 inhibitors have the potential to be effective anti-cancer agents that, when used in combination, will potentiate the cytotoxicity of a wide range of other chemotherapeutic agents *via* conferring synthetic lethality. In this scenario, CDK1 inhibitors with better specificity and pharmacokinetic properties seem to be a promising avenue to pursue cancer treatment.

In conclusion, CDK1 is required for efficient DNA replication, which adds to the functional versatility of CDK1. CDK1 inhibition disrupts normal DNA replication progression and causes DNA damage, which in turns imperils genomic stability. The requirement of CDK1 for DNA replication and genome stability maintenance provides a new target that can be exploited to achieve a better efficacy of combined therapies against human cancers.

## MATERIALS AND METHODS

### Cell culture

All cell lines were cultured in DMEM medium (Hyclone; Thermofisher) supplemented with 10% fetal bovine serum (Gibco) at 37°C under an atmosphere containing 5% carbon dioxide.

### Antibodies and agents

Primary antibodies used for Western blot and immunofluorescence were as follows: mouse anti-γH2AX (05-636, Millipore, 1:1000 for immunofluorescence), mouse anti-53BP1 (612523, BD Transduction Laboratories, 1:500 for immunofluorescence), mouse anti-RPA23 (ab2175, Abcam, 1:1000 for Western blot ), rabbit anti-phospho-RPA (ab109394, Abcam, 1:50000 for Western blot ), and mouse anti-β-actin (E021020-01, Earthox, 1:1000 for Western blot). Chemical agents used were as follows: RO3306 (Selleck), CDK2 inhibitor II (Selleck), Aphidicolin (Sigma), camptothecin (Selleck), Cisplatin (Selleck), and Olaparib (Selleck).

### Immunofluorescence and fluorescence microscopy

For quantification of nuclear foci, cells were seeded in 96-well plates and treated as indicated before fixation in 4% formaldehyde containing 0.1% Triton X-100 (Sigma) at room temperature (RT) for 15 minutes. Fixed samples were permeabilized and blocked for at least 1 hour (with 0.5% Triton X-100 and 3% BSA in PBS) or stored at 4°C until use. The samples were incubated with indicated primary antibodies at 4°C overnight. The incubation with secondary antibodies conjugated to Alexa Fluor 488 or 555 (Life Technologies) was performed at RT for 1 hour followed by staining with DAPI. For quantification of γH2AX foci in replicating and non-replicating cells, cultures were pulse-labeled with EdU for 1 hour before harvesting to identify replicating cells. After fixation, cells were first stained for EdU using the Click-iT-EdU kit (C10337, Invitrogen), according to the manufacturer’s protocol, and then standard immunostaining for γH2AX was performed. Images were visualized using an automated Nikon Eclipse Ni microscope with Nikon Elements software (Nikon Instruments) and captured using a 20× objective. For each sample, at least 1,000 cells from 3 random views are imaged and analyzed. The position of each cell nucleus was defined in the DAPI channel, and the number of nuclear γH2AX foci that co-localized or not with an EdU focus was counted using Nikon Elements software.

To perform single cell-based co-localization analysis, cells were seeded on coverslips and incubated with 10 μM EdU for 1 hour to label replication sites before harvesting. After fixation in 4% formaldehyde, cells were permeabilized and blocked as described above. EdU was stained using the Click-iT-EdU kit (C10337, Invitrogen), according to the manufacturer’s protocol. Cells were then stained for γH2AX (05-636, Millipore, 1:1000) and incubated with secondary antibody conjugated to Alexa Fluor 555 (Life technologies). Images were acquired on the Nikon Eclipse Ni microscope with Nikon Elements software (Nikon Instruments) using a 100× oil-immersion objective. The percentage of γH2AX foci colocalizing with EdU foci was calculated using Nikon Elements software.

### Western blot analysis

Cells were lysed in ice-cold RIPA buffer (50mM Tris-HCl, pH 7.4, 150 mM NaCl, 1% NP-40, 0.5% sodium deoxycholate, 0.1% SDS (Sigma)) supplemented with proteinase inhibitors (2 μg/ml leupeptin, 2 μg/ml aprotinin, 1 mM PMSF (Sigma)) and Phospho-stop cocktail (Roche). Cell lysates were sonicated at 80% amplitude for 10 seconds and then cleared by centrifugation at 13000 rpm for 15 minutes. Total protein content was measured for each sample using the BCA assay (Pierce) before 5× loading buffer (bromophenol blue (0.25%), glycerol (50%), SDS (sodium dodecyl sulfate; 10%), Tris-Cl (0.25 M, pH 6.8), mercaptoethanol (3.6%)) was added and protein samples were boiled at 95°C for 10 minutes. Equal amounts of protein were loaded and separated on 8−15% SDS-PAGE gels. Proteins were then transferred onto PVDF membranes (Millipore) using wet transfer with transfer buffer (25 mM Tris base, 189 mM glycine, 20% methanol) at 4°C. Membranes were washed in PBST and blocked with 5% non-fat milk in TBST for 1 hour, followed by sequential incubation with primary and secondary antibodies. Signals were detected using an Odyssey Clx membrane scanner.

### DNA fiber assays

Cells were grown in 6-well plates until they were 70% confluent. Before harvesting, cells were sequentially pulse-labeled with 25 µM CldU (Sigma) and 250 µM IdU (Sigma) for 10 minutes. Cells were then collected using trypsin EDTA and were resuspended in cold PBS at about 5×10^5^/ml. To spread the DNA fibers, 2 µl of cell suspension was pipetted on the slide and incubated for 5 minutes, and then gently mixed with 7 l of lysis buffer (200 mM Tris-HCl, pH 7.5, 50 mM EDTA, 0.5% SDS) before incubation for another 2 minutes. Slides were then tilted at an angle of 15°C to let the lysis buffer run down the slide and allow the DNA fibers to be stretched. The slides were then air-dried followed by fixation in methanol and acetic acid (3:1). The slides were rehydrated in PBS and denatured in 2.5M HCl for 80 minutes. After rinsing with PBS, slides were blocked with 1% BSA in PBS containing 0.1% Tween 20, and then incubated with mouse anti-BrdU for IdU (#347580, Becton Dickinson, 1:200) together with rat anti-BrdU for CldU (ab6326, Abcam, 1:1000) for 2 hours at RT. Primary antibodies were visualized using goat anti-mouse secondary antibody conjugated to Alexa Fluor 488 (Life technologies) and goat anti-rat secondary antibody conjugated to Alexa Fluor 594 (Abcam) for 1 hour at RT. Slides were then washed and mounted in mounting medium (Fluoromount-G^®^, Southern Biotech). Images were captured on the Nikon Eclipse Ni microscope with Nikon Elements software (Nikon Instruments) using a 100× oil-immersion objective. Fiber length measurement and replication structure fractionation were performed using Nikon Elements software. Tract length (red-green or green-red tracts) was converted to kilobases according to the conversion factor 1 µm = 2.59 kb, and then divided by the incubation time with the nucleoside analog to calculate fork speed. For each experiment, at least 500 replication structures were counted.

### EdU incorporation

To analyze EdU incorporation using flow cytometry, cells were grown in 6-well plates and EdU (10 µM) was added to the medium 10 minutes before harvesting. The cells were trypsinized, harvested, and then fixed in a combined fixation-permeabilization buffer (00-5123-43, concentrate, 00-5523-56 diluent, eBioscience) at 4°C for 1 hour, before washing in permeabilization buffer. Cells were harvested by centrifugation and incubated for 30 minutes with Click iT cocktail prepared according to the manufacturer’s protocol. After washing once with PBS, cells were resuspended in PBS for EdU detection on a Cytoflex flow cytometer (Beckman), 10,000 cells were collected and analyzed. For examination of EdU incorporation using microscopy, cells were seeded on coverslips and pulse-labeled with EdU before being fixed and permeabilized as described above. EdU was stained according to the manufacturer’s instructions and detected on the Nikon Eclipse Ni microscope. For each sample, at least 1,000 cells from 3 random views were analyzed.

### Analysis of micronuclei and chromatin bridges

Cells were grown in a 100-mm culture dish and were treated with DMSO or 7 µM of RO3306 for 16 hours. For the DMSO-treated group, mitotic cells were shaken off directly and reseeded onto coverslips followed by fixation in 4% formaldehyde 15 minutes later. For the RO3306 treated group, cells were first washed 3 times with warm PBS to rinse away the inhibitor, and then cells were released into the pre-warmed fresh medium and incubated for 5 minutes to allow transition into early M phase. Mitotic cells were shaken off and reseeded as described above. To obtain anaphase cells for chromatin bridge quantification, cells were fixed 50 minutes after reseeding. For quantification of micronuclei in G1 daughter cells, samples were harvested 90 minutes after reseeding. Nuclei were visualized using DAPI staining and mounted using the mounting medium. For each experiment, at least 1000 nuclei were counted.

### Clonogenic assays

Clonogenic assays were performed with 500 cells per well in 6-well plates. After adhesion, cells were treated with increasing doses of different drugs in the presence or absence of 1 µM RO3306. Drugs were left in the growth medium for the entire experimental period. Colony numbers were counted 10−12 days after initial drug addition by staining with crystal violet (0.5% in PBS, w/v). The surviving fraction of cells was calculated and presented relative to the DMSO-treated control cell group.

### Statistics

All data were analyzed using GraphPad Prism 7.0. A Mann-Whitney test was used as a non-parametric test. For two-group comparisons, a two-tailed *t*-test was performed when the data conformed to a normal distribution. A one-way ANOVA test was applied when comparing more than two groups followed by Dunnett’s multiple comparison post-test. For grouped analysis, a two-way ANOVA test followed by a Sidak multiple comparison post-test was used. Data were considered as significant at *p* < 0.05.

## SUPPLEMENTARY MATERIALS FIGURES



## Abbreviations

CDK1i: CDK1 inhibitor
DDR: DNA damage response
DMEM: Dulbecco minimum essential medium
DMSO: dimethyl sulfoxide
DSB: double-strand breaks
EdU: 5-ethynyl-2´-deoxyuridine
HR: homologous recombination
PBS: phosphate Buffered Solutions
SDS: sodium dodecyl sulfate

## References

[1] Petrone A, Adamo ME, Cheng C, Kettenbach AN (2016). Identification of Candidate Cyclin-dependent kinase 1 (Cdk1) Substrates in Mitosis by Quantitative Phosphoproteomics. Mol Cell Proteomics.

[2] Bashir T, Pagano M (2005). Cdk1: the dominant sibling of Cdk2. Nature Cell Biology.

[3] Gautier J, Minshull J, Lohka M, Glotzer M, Hunt T, Maller JL (1990). Cyclin is a component of maturation-promoting factor from Xenopus. Cell.

[4] Sanchez I, Dynlacht BD (2005). New insights into cyclins, CDKs, and cell cycle control. Semin Cell Dev Biol.

[5] Satyanarayana A, Kaldis P (2009). Mammalian cell-cycle regulation: several Cdks, numerous cyclins and diverse compensatory mechanisms. Oncogene.

[6] Lim S, Kaldis P (2013). Cdks, cyclins and CKIs: roles beyond cell cycle regulation. Development.

[7] Diril MK, Ratnacaram CK, Padmakumar VC, Du T, Wasser M, Coppola V, Tessarollo L, Kaldis P (2012). Cyclin-dependent kinase 1 (Cdk1) is essential for cell division and suppression of DNA re-replication but not for liver regeneration. Proc Natl Acad Sci U S A.

[8] Martin A, Odajima J, Hunt SL, Dubus P, Ortega S, Malumbres M, Barbacid M (2005). Cdk2 is dispensable for cell cycle inhibition and tumor suppression mediated by p27(Kip1) and p21(Cip1). Cancer Cell.

[9] Aleem E, Kiyokawa H, Kaldis P (2005). Cdc2-cyclin E complexes regulate the G1/S phase transition. Nat Cell Biol.

[10] Albrecht JH, Hoffman JS, Kren BT, Steer CJ (1993). Cyclin and cyclin-dependent kinase 1 mRNA expression in models of regenerating liver and human liver diseases. Am J Physiol.

[11] Taneera J, Fadista J, Ahlqvist E, Zhang M, Wierup N, Renstrom E, Groop L (2013). Expression profiling of cell cycle genes in human pancreatic islets with and without type 2 diabetes. Mol Cell Endocrinol.

[12] Wang Z, Slipicevic A, Forsund M, Trope CG, Nesland JM, Holm R (2015). Expression of CDK1(Tyr15), pCDK1(Thr161), Cyclin B1 (total) and pCyclin B1(Ser126) in vulvar squamous cell carcinoma and their relations with clinicopatological features and prognosis. PLoS One.

[13] Hochegger H, Dejsuphong D, Sonoda E, Saberi A, Rajendra E, Kirk J, Hunt T, Takeda S (2007). An essential role for Cdk1 in S phase control is revealed *via* chemical genetics in vertebrate cells. The Journal of Cell Biology.

[14] Garnier D, Loyer P, Ribault C, Guguen-Guillouzo C, Corlu A (2009). Cyclin-dependent kinase 1 plays a critical role in DNA replication control during rat liver regeneration. Hepatology (Baltimore, Md).

[15] Johnson N, Cai D, Kennedy RD, Pathania S, Arora M, Li YC, D’Andrea AD, Parvin JD, Shapiro GI (2009). Cdk1 participates in BRCA1-dependent S phase checkpoint control in response to DNA damage. Mol Cell.

[16] Johnson N, Li YC, Walton ZE, Cheng KA, Li D, Rodig SJ, Moreau LA, Unitt C, Bronson RT, Thomas HD, Newell DR, D’Andrea AD, Curtin NJ (2011). Compromised CDK1 activity sensitizes BRCA-proficient cancers to PARP inhibition. Nature Medicine.

[17] Ira G, Pellicioli A, Balijja A, Wang X, Fiorani S, Carotenuto W, Liberi G, Bressan D, Wan L, Hollingsworth NM, Haber JE, Foiani M (2004). DNA end resection, homologous recombination and DNA damage checkpoint activation require CDK1. Nature.

[18] Simoneau A, Robellet X, Ladouceur AM, D’Amours D (2014). Cdk1-dependent regulation of the Mre11 complex couples DNA repair pathways to cell cycle progression. Cell Cycle (Georgetown, Tex).

[19] Pierre H, Helen W, Clara CR (2014). Miguel Godinho F, Aidan JD. Cdk1 Restrains NHEJ through Phosphorylation of XRCC4-like Factor Xlf1. Cell Reports.

[20] Chow JP, Poon RY, Ma HT (2011). Inhibitory phosphorylation of cyclin-dependent kinase 1 as a compensatory mechanism for mitosis exit. Molecular and Cellular Biology.

[21] Chen X, Niu H, Chung WH, Zhu Z, Papusha A, Shim EY, Lee SE, Sung P, Ira G (2011). Cell cycle regulation of DNA double-strand break end resection by Cdk1-dependent Dna2 phosphorylation. Nature Structural & Molecular Biology.

[22] Vassilev LT, Tovar C, Chen S, Knezevic D, Zhao X, Sun H, Heimbrook DC, Chen L (2006). Selective small-molecule inhibitor reveals critical mitotic functions of human CDK1. Proc Natl Acad Sci U S A.

[23] Al Abo M, Dejsuphong D, Hirota K, Yonetani Y, Yamazoe M, Kurumizaka H, Takeda S (2014). Compensatory functions and interdependency of the DNA-binding domain of BRCA2 with the BRCA1-PALB2-BRCA2 complex. Cancer Res.

[24] Zhang F, Ma J, Wu J, Ye L, Cai H, Xia B, Yu X (2009). PALB2 links BRCA1 and BRCA2 in the DNA-damage response. Curr Biol.

[25] Schlacher K, Christ N, Siaud N, Egashira A, Wu H, Jasin M (2011). Double-strand break repair-independent role for BRCA2 in blocking stalled replication fork degradation by MRE11. Cell.

[26] Neelsen KJ, Lopes M (2015). Replication fork reversal in eukaryotes: from dead end to dynamic response. Nature Reviews Molecular Cell Biology.

[27] Lord CJ, Ashworth A (2016). BRCAness revisited. Nat Rev Cancer.

[28] Gaillard H, Garcia-Muse T, Aguilera A (2015). Replication stress and cancer. Nat Rev Cancer.

[29] Franzolin E, Pontarin G, Rampazzo C, Miazzi C, Ferraro P, Palumbo E, Reichard P, Bianchi V (2013). The deoxynucleotide triphosphohydrolase SAMHD1 is a major regulator of DNA precursor pools in mammalian cells. Proc Natl Acad Sci U S A.

[30] Alexandra C, Benjamin D, Ana Luiza Chaves V, Nadine L, Monsef B (2013). Phosphorylation of SAMHD1 by cyclin A2/CDK1 regulates its restriction activity toward HIV-1. Cell Reports.

[31] Dobbelstein M, Sorensen CS (2015). Exploiting replicative stress to treat cancer. Nat Rev Drug Discov.

[32] Zeman MK, Cimprich KA (2014). Causes and consequences of replication stress. Nat Cell Biol.

