# Supplementary material for: CDK1 promotes nascent DNA synthesis and induces resistance of cancer cells to DNA-damaging therapeutic agents
Source: Oncotarget. 2017 Oct 10;8(53):90662–73. doi: 10.18632/oncotarget.21730 (PMC5710876; doi:10.18632/oncotarget.21730)
Supplement: Supplementary file 1 [file oncotarget-08-90662-s001.pdf]

CDK1 promotes nascent DNA synthesis and induces resistance of cancer cells to DNA-damaging therapeutic agents

SUPPLEMENTARY MATERIALS

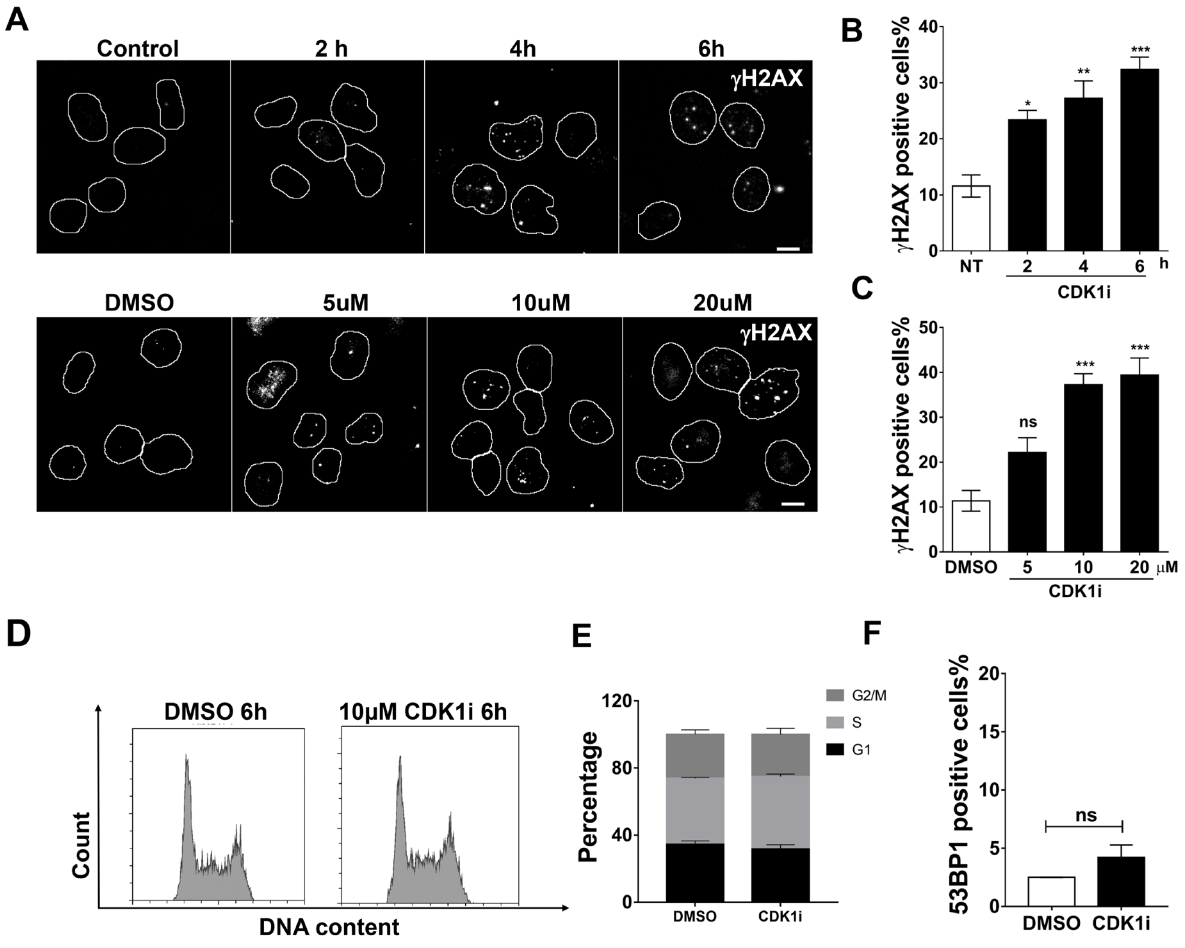

Supplementary Figure 1: CDK1 inhibition induces a DNA damage response independently of cell type, cell cycle progression, and DSB repair. (**A**) Representative images of H2AX foci formation in A549 cells treated with 10 μM CDK1i for the indicated times (upper panel) or with increasing concentrations of CDK1i for 4 hours (lower panel); scale bar, 20 μm; (**B**–**C**) Quantification of the data from the upper and lower panels of A, respectively. Cells with over 3 γH2AX foci were counted as positive cells. Results are presented as the mean±SEM from three independent experiments. (**D**) Representative FACS-based analysis of cell cycle distribution of U2OS cells treated with DMSO or CDK1i for 6 hours. (**E**) Quantification of D. (**F**) Quantification of 53BP1 foci in U2OS cells after a 6-hour treatment with DMSO or CDK1i. For B–C, data are pooled from three independent experiments and shown as means±SEM, the significance was determined using a one-way ANOVA followed by Dunnett's multiple comparison post-test. For E, data are means±SEM of three replicates from a representative experiment, a two-way ANOVA followed by a Sidak multiple comparison post-test was used. For F, a Student's *t*-test was used to calculate p values *, $p<0.05$; **, $p<0.01$; ***, $p<0.001$; ns, not significant.

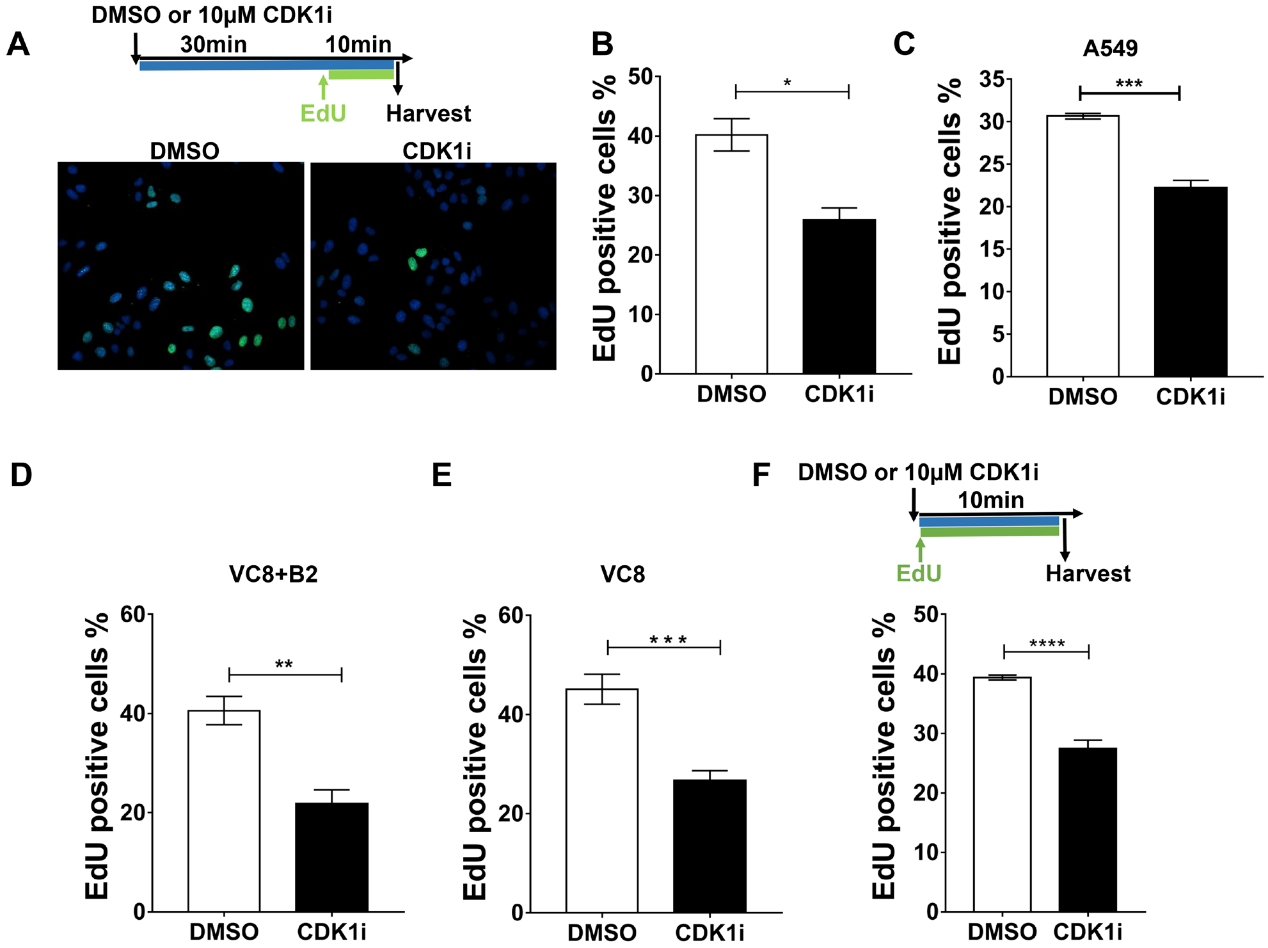

Supplementary Figure 2: CDK1 promotes nascent DNA synthesis in a manner that is independent of cell type and BRCA2 status. (**A**) Experimental scheme (upper panel) and representative immunofluorescence images (lower panel) of EdU incorporation in U2OS cells treated as indicated. (**B**) Quantification of EdU positive cells from A. (**C**) Quantification of EdU-positive cells detected by FACS in A549 cells subjected to treatment as in a. (**D–E**) Quantification of cells positive for EdU determined by FACS in VC8 cells supplemented with BRCA2 (VC8+B2) (D) and VC8 cells lacking BRCA2 (E). (F) Quantification of EdU-positive U2OS cells treated as indicated in the experimental scheme shown above. Overall, data are presented as the means±SEM from three independent experiments in B–E and from five independent experiments in F. Significance was determined using Student's *t*-tests. *, p<0.05; **, p<0.01; ***, p<0.001; ****, p<0.0001; ns, not significant.

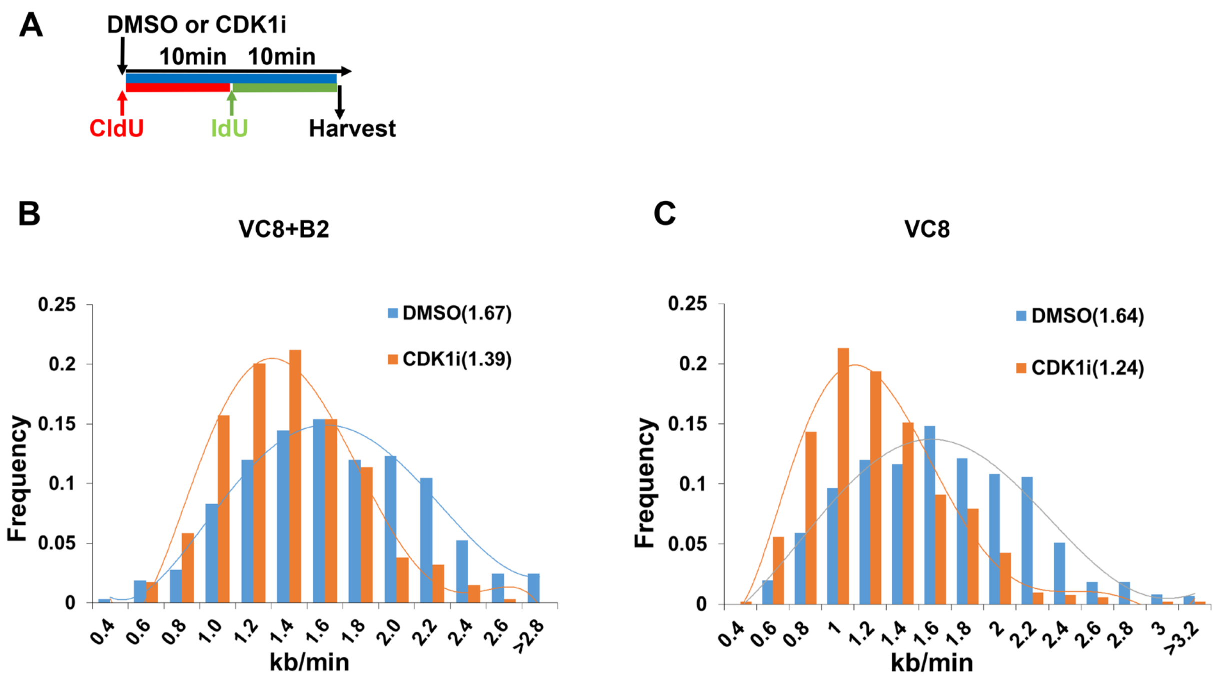

Supplementary Figure 3: CDK1 is required for efficient DNA replication fork progression in both BRCA2-proficient and -deficient cells. Replication fork analysis of VC8+B2 and VC8 cells pulsed with the thymidine analogs, chlorodeoxyuridine (CldU) and iododeoxyuridine (IdU), in the presence of DMSO or CDK1i. Experimental scheme (**A**), fork speed quantification of VC8+B2 cells (**B**), and fork speed quantification of VC8 cells (**C**). Numbers in parentheses are the mean fork speed.
